# Canine Distemper: Its Causes, Symptoms, Sequels, and Treatment

**Published:** 1895-02

**Authors:** E. Wadams


### CANINE DISTEMPER: ITS CAUSES, SYMPTOMS, SEQUELS, AND TREATMENT.

BY E. WADAMS, V.S.

MR. PRESIDENT AND GENTLEMEN: The subject I have chosen for my essay is one, I think, much overlooked by the veterinary profession, for the simple reason that the majority of practitioners consider "canine practice" of small importance, and not sufficiently remunerative for the time and trouble expended; but, be that as it may, it is a source of considerable income to me, for I find that men who own dogs valuable enough to treat, do what they can cheerfully for their pets, and are willing to pay reasonably for the effort.

At one time I was inclined to believe, from the numerous forms distemper assumes, that there was really no such distinct disease, but that a variety of complaints had been ignorantly jumbled together under a term which affords no distinguishing mark by which to test its separate existence. But closer observation has enabled me, I think, to seize the clue which explains all these apparent discrepancies, and also leads the way to the scientific treatment of this sometimes unmanageable disorder.

I began my investigations by considering what symptoms invariably attend upon distemper. At first I was completely puzzled, for I found in one series of cases nothing but cough and discharge at the nose and eyes, in another these symptoms were absent, and their place supplied by head symptoms and fits, whilst again in the third set, the dogs all suffered from diarrhœa with discharges of blood, and had neither head nor chest symptoms in any stage of the disease. In all these cases, however, I found there was fever, and that of a peculiar kind. This was not like the ordinary feverishness to which dogs are subject, and which attends upon almost all their acute attacks, but of a low typhoid character, with great exhaustion, loss of appetite, and in the latter stages a collection of brown fur, or as it is called in human medicine "sordes," about the teeth.

I have come to the conclusion that a protracted case of distemper is nothing more or less than typhus fever, and in that word typhus might be found the key to the anomalies so puzzling to all inquirers into the nature of the malady.

It seems strange that such men as Drs. Jenner, James Woodruff, Hill, Forrest, and others, did not think of this fact; but if so, have failed to mention it. Their experience in vaccination proved a failure, and this is the universal opinion of all modern professional men who have given it a trial. Everyone, however, must recollect numerous instances in which a simple clue once seized will explain away all differences or difficulties, except the one astonishing fact of this simple clue not having long ago been detected.

The points of similarity between distemper in dogs and typhus fever in man are so strongly marked that a treatise upon the latter is all that is wanted to enable anyone who understands medical terms or phrases to treat distemper, or, as I call it, typhus fever, with the greatest probability of success.

I know full well that this disease is looked upon by many with contempt as a loathsome disease, but we are here to treat all domestic animals, and why should the ailments of our faithful friend the dog be omitted? His care and treatment belong to our profession and demand attention, and in years to come the treatment of the true friend, the dog, will be sought by the professional man instead of ignored as unworthy of his attention.

How many practitioners are there who do not despise the dog as a patient, and hate to be called upon to treat him? but for my part give me a man who loves or appreciates the value of his dog, and I can invariably satisfy its owner, replenish my exchequer, and do some good to the poor though despised dumb brute.

The old adage still remains, and that is—'tis only a dog—and poor old Watch, Rover, or Spot, is left to die or get well as the case may be, starve or freeze, after years of faithful service.

Symptoms. I define distemper in the dog as a fever of a nature similar to typhus, and always followed by these symptoms:

There are first dulness and restlessness, with partial loss of appetite, heat and dryness of the nose, rapid but feeble pulse, and dull eye, the white of which is generally streaked with dark-red bloodvessels, and the dog seems to be extremely sensitive to cold. The urine is scanty and high-colored; considerable thirst; the movements of the bowels are either costive or loose, if the latter, often of pitchy color; the flesh and strength fail in a remarkably rapid manner, so that the dog is unable to stand inside of a week; the appetite by that time is entirely lost, and frequently the food which is given by force is returned or speedily passed through the bowels unchanged. The respiration generally becomes quicker than natural, though this symptom is not so universal as others I have mentioned. In fact, all the processes of respiration, circulation, digestion, and secretion are disturbed to a remarkable degree, and in this I believe is the essence of the disease, and the healthy return of these functions is always an indication of convalescence; the blood is no longer depurated properly by the agency of the lungs, liver, and kidneys, nor renewed by healthy materials, the result of good digestion.

The circulation, consequently, is imperfectly carried on, and the brain is supplied with impure blood which is full of matters acting as a poison to it, and as a consequence death often takes place at an early date from a fit. Whatever secretions are affected are vitiated in quality and altered in quantity, sometimes being almost stopped, occasioning the dry husky cough and constipation of the early stage; and sometimes in excess, where you have the profuse offensive discharge from the nose and bronchi, or the pitchy discharges from the intestines, which accompany the progress of the disease. Such are the general and characteristic symptoms of the disease.

The local complications involved in this complaint may be enumerated as follows: 1st. The affections of the chest. 2d. Of the liver, stomach, and bowels. 3d. The kidneys. 4th. Of the brain and nervous system. 5th. Of the skin, each trouble being represented by the usual phenomena pertaining to said organs.

Etiology. The most common cause of distemper is, no doubt, infection; but it is occasionally epizootic, and then most probably depends upon some condition of the air. But it also seems to be the natural tendency of all debilitating diseases in young dogs to run on into this typhoid type.

Whether the disease is common catarrh or influenza, or inflamed liver or lungs, or even the irritative fever of worms or teething, in all these cases the tendency seems to be to put on the form of low typhus, and this is especially the case when dogs are ill-fed or crowded together in poorly-ventilated kennels, or, in fact, submitted to any of the ordinary predisposing causes of typhus fever in the human subject. And this disease once established, soon spreads to all within the reach of its influence. The infection seems to be carried with them by dogs after their convalescence. I have frequently known a severe attack supervene upon the contact with a puppy in the slips which was sufficiently recovered to run, though, of course, not in a fit state for such an exertion.

The kennel also seems to retain the infection for a long time, it being most probably absorbed in the urine, etc.

The walls, etc., should be well washed with chloride of lime or some other disinfecting fluid. After the dogs are removed some time should elapse before others are placed in them. Dogs seem to be particularly liable to distemper when approaching maturity, the greater number being attacked between nine and eighteen months old, but in the typhus fever of man it is not confined to that age; the suckling and the old dog are also open to its attack, though in a much less degree.

As in the human being, one attack generally, though not always, preserves the individual from another attack.

Indeed, the laws of contagion and the statistics of the two diseases are precisely the same, making allowance, of course, for the difference in the time of coming to maturity, and reckoning age in the dog by months and in man by years, so that a dog twenty-one months old may be considered as mature as a man of twenty-one years of age.

Period of incubation. This disease, like typhus fever, takes from ten days to two weeks after infection before making its appearance.

Duration. Distemper varies in the length of attack from a few days to two or even three months; but the average period, like that of typhus fever, is twenty-one days.

Diagnosis. In its mildest form distemper is very often mistaken for ephemeral fever or influenza. Severe distemper is most likely to be confounded with hydrophobia, from which it may be distinguished either by the absence of all aberration of intellect or by the difference of the form of its manifestations. In distemper there is no restlessness, or, at all events, only a tendency to change the posture, while the rabid dog is always on the watch, and eyes every one with a wild, suspicious gaze. The distempered dog is certainly nervous, but only from fear; while the hydrophobic dog knows neither fear nor pain, and will also resist to the death any threats or actual punishment.

If the brain is much affected in distemper, there is almost always a fit or paralysis, neither of which occurs in hydrophobia; and lastly, the distempered dog eats nothing, while the hydrophobic animal has a depraved appetite and devours any filthy material that comes in his way.

Distemper is liable to be confounded with any of the inflammatory diseases which attack the brain, lungs, liver, stomach, or kidneys; but in simple inflammation of any of these organs there is not the extreme and speedy emaciation and loss of strength noticed always in distemper.

Treatment. The management and remedies used in this disease must vary with the stages of the malady, and must necessarily be considered under four heads, viz.: The periods of incipiency, reaction, typhoid, and convalescence. The incipient stage is, I am sure, treated with more success in the following manner than any other.

Put your puppy or dog, as the case may be, in a comfortable, warm, well-ventilated place, avoiding, of course, a damp or dark cellar; a good dry bed and clothing if in a cold climate. Give a puppy ten months old the following:

| | |
|---|---|
| ℞.—Hyd. Chlor. Mite . . . . . . . | 2 grains. |
| Pil. Rhei Comp. . . . . . . . | 1 drachm. |
| Sodæ Bicarb. . . . . . . . . | 30 grains. |

M. ft. pulv. Put in six capsules.
Sig.—Give one every two hours until all are taken.

At the same time put ten grains of the hyposulphite of soda to each pint of water the dog takes. Feed on boiled milk, soda crackers, and a little well-cooked mutton, it being easier digested. The stomach will bear more mutton than any other meat. Many men advise oatmeal, but from an extensive experience, both in treating this disease and many others, I find oatmeal too loosening unless combined with a sufficiency of wheat flour, and as the bowels have generally a tendency to relaxation, we cannot be too careful, but I must here remark that to a healthy dog Scotch oatmeal is his greatest support.

The period of reaction, I may say, commences the moment a local symptom shows itself, and it is at this stage that the greatest difficulty in the treatment arises. The chief complications that draw our attention now are the chest, the abdomen, and the head, the skin complications being of minor importance, except as a sign of the severity of the attack.

We will now take up the chest uncomplicated with any affection of the bowels.

Now, gentlemen, with all due respect to my superiors, I am antagonistic to all. Drs. Stonehage, J. W. Hill, Jenner, and numerous others claim tartar emetic their sheet-anchor. I claim it is a dead letter.

In chest complications I want to lower the temperature and keep up the strength, owing to the debilitating influence of the disease. To dose we must use our knowledge of materia medica, and this is my conclusion after considerable experience in the treatment of this disease, with a fair knowledge of the above-named book.

I take my temperature, and if I find a high one, I give a toxical dose of quinine, say ten grains, put in a hot bath and rub dry, then blanket, and if not relieved in eight hours I blister the sides with cantharides, muzzling the dog at the time; then I give the following prescription, being always particular in its administration.

| | |
|---|---|
| ℞.—Pulv. Digitalis . . . . . . . | 6 grains. |
| Pulv. Pot. Nitratas . . . . . | 1½ drachm. |
| Ext. Glycyrrhiza . . . . . . | 1 drachm. |
| Syrup . . . . . . . . . | q.s. |

M. F. Mass et divide in capsules No. xii.
Sig.—Give one every four hours.

If the animal does not improve on this treatment, and seems to lack recuperative powers, I begin a stimulating treatment, feeding beef tea, mutton broth, and raw eggs beaten up with milk.

The abdomen is generally the seat of severe intestinal disorders, the principal being a diarrhœa accompanied with pitchy discharges. I invariably treat this complication with small quantities of calomel, one-sixth of a grain, prepared chalk, ten grains, or subnitrate of bismuth and turpentine emulsions, always remembering if any intestinal disturbance is due to intestinal parasites, remove them as soon as possible, using the mildest remedies on account of the irritable conditions of the alimentary canal, areca-nut for the tænia family, and oil of woodworm for the lumbricoids.

The head symptoms are generally a severe complication, especially if attended with chorea. I put a seton in the back of the neck, put tr. iodine and tr. cantharides along the spine, every other day for two applications, and give internally the following:

| | |
|---|---|
| ℞.—Strychnia Sulph . . . . . . . | 1 grain. |
| Ext. Gent . . . . . . . . . | q.s. |

M. F. and Mass. Divide in fifty pills and put in capsules, No. 50.
Sig.—Give one night and morning after feeding, gradually increasing the dose until four a day can be taken.

Typhoid Stages. Watch your patient carefully, as this is the stage which I believe indicates the essence of the disease known as distemper, and unless the dog has been evidently submitted to the contagion, it cannot be pronounced to have had the distemper.

I give the following tonic three times daily: Quinine, one grain; Portwine, one-half ounce, or cod-liver oil, one-half ounce; and tr. chloride of iron, ten minims, controlling the bowels with subnitrate of bismuth, ten grains, and sulphate of morphia, one-fourth of a grain every four or six hours as occasion requires.

Use injections of starch, always using such food as will be easily digested, and be sufficiently nourishing to maintain the strength of the patient.

Now, Mr. President and gentlemen, I thank you kindly for your patience and attention, and I sincerely hope that this essay may elicit some discussion. The subject, I am aware, is obnoxious to many, but in the future will undoubtedly benefit the whole profession, for, as the dog is bred, so is the requirements of the veterinarian in demand. This simple essay, although not so technical, pathological, or flowery as some, is my own experience based upon facts. I hope this may hold up support or in some way benefit the poor but faithful companion of man, the dog.

